# Causal relationships between milk quality and coagulation properties in Italian Holstein-Friesian dairy cattle

**DOI:** 10.1186/s12711-015-0123-7

**Published:** 2015-05-13

**Authors:** Francesco Tiezzi, Bruno D Valente, Martino Cassandro, Christian Maltecca

**Affiliations:** Department of Animal Science, North Carolina State University, Raleigh, NC 27695 USA; Department of Animal Science, University of Wisconsin, Madison, WI 53706 USA; Department of Agronomy, Food, Natural Resources, Animals and Environment, University of Padova, 35020 Legnaro, (PD) Italy

## Abstract

**Background:**

Recently, selection for milk technological traits was initiated in the Italian dairy cattle industry based on direct measures of milk coagulation properties (MCP) such as rennet coagulation time (RCT) and curd firmness 30 min after rennet addition (a_30_) and on some traditional milk quality traits that are used as predictors, such as somatic cell score (SCS) and casein percentage (CAS). The aim of this study was to shed light on the causal relationships between traditional milk quality traits and MCP. Different structural equation models that included causal effects of SCS and CAS on RCT and a_30_ and of RCT on a_30_ were implemented in a Bayesian framework.

**Results:**

Our results indicate a non-zero magnitude of the causal relationships between the traits studied. Causal effects of SCS and CAS on RCT and a_30_ were observed, which suggests that the relationship between milk coagulation ability and traditional milk quality traits depends more on phenotypic causal pathways than directly on common genetic influence. While RCT does not seem to be largely controlled by SCS and CAS, some of the variation in a_30_ depends on the phenotypes of these traits. However, a_30_ depends heavily on coagulation time. Our results also indicate that, when direct effects of SCS, CAS and RCT are considered simultaneously, most of the overall genetic variability of a_30_ is mediated by other traits.

**Conclusions:**

This study suggests that selection for RCT and a_30_ should not be performed on correlated traits such as SCS or CAS but on direct measures because the ability of milk to coagulate is improved through the causal effect that the former play on the latter, rather than from a common source of genetic variation. Breaking the causal link (e.g. standardizing SCS or CAS before the milk is processed into cheese) would reduce the impact of the improvement due to selective breeding. Since a_30_ depends heavily on RCT, the relative emphasis that is put on this trait should be reconsidered and weighted for the fact that the pure measure of a_30_ almost double-counts RCT.

**Electronic supplementary material:**

The online version of this article (doi:10.1186/s12711-015-0123-7) contains supplementary material, which is available to authorized users.

## Background

In recent years, increasing efforts have been made to enhance efficiency in the Italian dairy industry and dairy cattle breeding organizations have started selecting for a wide range of novel traits. Milk coagulation properties (MCP) have been included in the data recording system and breeding values are routinely produced for Italian Holstein bulls [1]. Milk coagulation properties, namely rennet coagulation time (RCT) and curd firmness after 30 min from rennet addition (a_30_), have been shown to be good predictors of milk technological quality and cheese yield [2-4], which are key factors in dairy industries where most of the milk produced is processed into cheese. In particular, a_30_ is the trait that has the strongest impact on Grana Padano cheese processing [4].

Generally, selection for RCT and a_30_ is based either on correlated traits such as somatic cell score (SCS), fat, protein and casein percentages [5-8] or on direct measures of RCT and a_30_ [1,9]. These two traits can vary in terms of curd firmness at different time points in the milk coagulation process and depend heavily on each other. This is inherent to the test used (see Bittante [10] and Bittante et al. [11] for a review of current knowledge), i.e. RCT (in min) measures the amount of time between rennet addition and the beginning of the coagulation process, whereas a_30_ measures curd firmness 30 min after rennet addition. The longer the milk takes to start coagulating, the softer the curd will be at the end of the test, and vice versa. Somatic cell score and milk casein percentage (CAS) are considered to affect RCT and a_30_ [12-14] and are correlated at the genetic level [5,15]. Pretto et al. [7] suggested that the genetic correlation that exists between SCS and CAS could be used as a predictor in breeding programs that focus on improving MCP.

The overall genetic effects that influence MCP are probably distributed into multiple causal paths: on the one hand, some genes may affect MCP directly, while, on the other hand, some genes may affect other milk quality parameters, which in turn affect the ability of milk to coagulate. Alternatively, a causal path that involves MCP may exist. For instance, a strong association between a_30_ and RCT could support a causal hypothesis that variability in a_30_ is mostly explained by the influence of RCT, while there is no strong direct genetic effect on a_30_ (i.e. they are independent). In other words, some genes may not strongly and directly affect both RCT and a_30_, but only RCT.

As discussed by [16], in the classical genetic evaluation scenario, breeding values of candidate individuals are predicted by fitting multiple trait models (MTM), which neglect the causal network that influences phenotypic traits. Structural equation models (SEM) [17-31] can help “dissect” the overall genetic effects expressed by MTM into distinct sources of genetic variation, by separating the common sources of variation that affect directly two or more traits in the system (e.g. the genetic correlation between SCS and RCT) from the causal effect that one phenotypic trait plays on the other (e.g. the causal effect of SCS on RCT). In addition, using non-intervened data (such as field data routinely collected for genetic evaluations), SEM would be able to predict genetic effects for scenarios for which interventions on the phenotype are performed [16]. For example, let us consider a scenario in which the goal is to predict the individuals’ genetic effects on RCT or a_30_ when milk quality traits are physically controlled (e.g., by filtering somatic cell load [32,33] or standardizing casein percentage [34,35]). Such a scenario will take only the individuals’ genetic effects on RCT and a_30_ into account, since the genetic influence mediated by SCS and CAS is blocked. Alternative approaches to assess the impact of SCS and CAS on MCP require that additional experimental records, this time under the given intervention, and then genetic effects based on these data can be predicted. Therefore, the genetic parameters and breeding values for a_30_ that are estimated with SEM result in interpretations that differ from those obtained with MTM. The breeding value of a given cow that is estimated with SEM for a_30_ indicates its genetic merit in terms of firmness of the milk produced that is not mediated by SCS and CAS and that cannot be obtained with standard MTM. Moreover, if indirect effects play a major role in the variation of a_30_ and if the traits through which the effect is mediated are in the selection index, it might not be necessary to consider a_30_ since that would represent redundant information.

The aim of this study was to: (1) infer the magnitude of the causal effects of two traditional milk quality traits (SCS and CAS) on MCP, (2) estimate the causal effect of RCT on a_30_ and the genetic variation in the latter when the causal effect of the former is removed, and (3) estimate genetic and phenotypic variation of a_30_ by taking the causal effect of all other traits into account, i.e. by assessing the magnitude of the variance components that would hold if some milk quality parameters are controlled.

## Methods

### Data collection and editing procedure

Routine assessment of MCP via mid-infrared spectroscopy began in September 2011 in the Veneto region of Italy [1,36]. Approximately 25 000 cows are currently under monthly routine control. A panel of traits is routinely assessed with Milko-Scan FT6000 (Foss Electric A/S, Hillerød, Denmark), including fat, protein and casein percentages, milk coagulation properties (such as RCT, curd firming time and a_30_) and fatty acid profile of milk. Milk somatic cell count (SCC) is determined with Cell Fossomatic 250.

For this study, we extracted data from the same dataset as in Tiezzi et al. [36]. Traditional milk quality parameters were chosen, i.e. SCS (as log-transformation of SCC) and CAS, while we used RCT and a_30_ as measures of MCP. We retained only the records from early-lactation (5 to 125 days in milk) on first-lactation cows in order to avoid accumulation of carry-over effects of deteriorated milk quality on coagulation properties (e.g. an identical decrease in SCS may have a different impact on RCT in early and late lactation because of the accumulation of the effect of SCS over lactation, such that late lactation RCT and a_30_ may be affected by early lactation SCS, late lactation SCS and their interaction). Therefore, data editing was similar to that in [36], except that only the records from early-lactation (5 to 125 days in milk) first-parity cows were considered. For statistical analysis, 8783 records collected on 3266 first-lactation Italian Holstein cows across the period from January to December 2012 were used for statistical analyses. Cows were sired by 128 AI bulls and reared in 309 herds.

### Statistical analysis

We fitted three SEM and a single MTM. The baseline MTM (**M0**) was as follows:

$$ \mathbf{M}0\ \left\{\begin{array}{c}\hfill {\mathbf{y}}_1=\mathbf{X}{\mathbf{b}}_1+{\mathbf{Z}}_{\mathbf{h}}{\mathbf{h}}_1+{\mathbf{Z}}_{\mathbf{p}}{\mathbf{p}}_1+{\mathbf{Z}}_{\mathbf{s}}{\mathbf{s}}_1+{\mathbf{e}}_1\hfill \\ {}\hfill {\mathbf{y}}_2=\mathbf{X}{\mathbf{b}}_2+{\mathbf{Z}}_{\mathbf{h}}{\mathbf{h}}_2+{\mathbf{Z}}_{\mathbf{p}}{\mathbf{p}}_2+{\mathbf{Z}}_{\mathbf{s}}{\mathbf{s}}_2+{\mathbf{e}}_2\hfill \\ {}\hfill {\mathbf{y}}_3=\mathbf{X}{\mathbf{b}}_3+{\mathbf{Z}}_{\mathbf{h}}{\mathbf{h}}_3+{\mathbf{Z}}_{\mathbf{p}}{\mathbf{p}}_3+{\mathbf{Z}}_{\mathbf{s}}{\mathbf{s}}_3+{\mathbf{e}}_3\hfill \\ {}\hfill {\mathbf{y}}_4=\mathbf{X}{\mathbf{b}}_4+{\mathbf{Z}}_{\mathbf{h}}{\mathbf{h}}_4+{\mathbf{Z}}_{\mathbf{p}}{\mathbf{p}}_4+{\mathbf{Z}}_{\mathbf{s}}{\mathbf{s}}_4+{\mathbf{e}}_4\hfill \end{array}\right., $$

where the index ‘*i*’ indicates correspondence to the i^th^ trait, **y**_**1,**_, **y**_**2**_, **y**_**3**_ and **y**_**4**_ are the vectors reporting the four traits (SCS, CAS, RCT and a_30_, considered in this order), **X** and **b**_**i**_ are the incidence matrix and the corresponding vector of fixed effects (intercept and four classes of stage of lactation, namely 5 to 34, 35 to 64, 65 to 94 and 95 to 125 days in milk), **Z**_**h**_ and **h**_**i**_ are the incidence matrix and corresponding vector of herd random effect (309 levels), **Z**_**p**_ and **p**_**i**_ are the incidence matrix and vector of cow permanent environmental random effect (3266 levels), **Z**_**s**_ and **s**_**i**_ are the incidence matrix and vector of sire random additive genetic effect (128 sires, 1254 total individuals in the sire-MGS pedigree), and **e**_**i**_ are random residuals.

**Figure 1:**
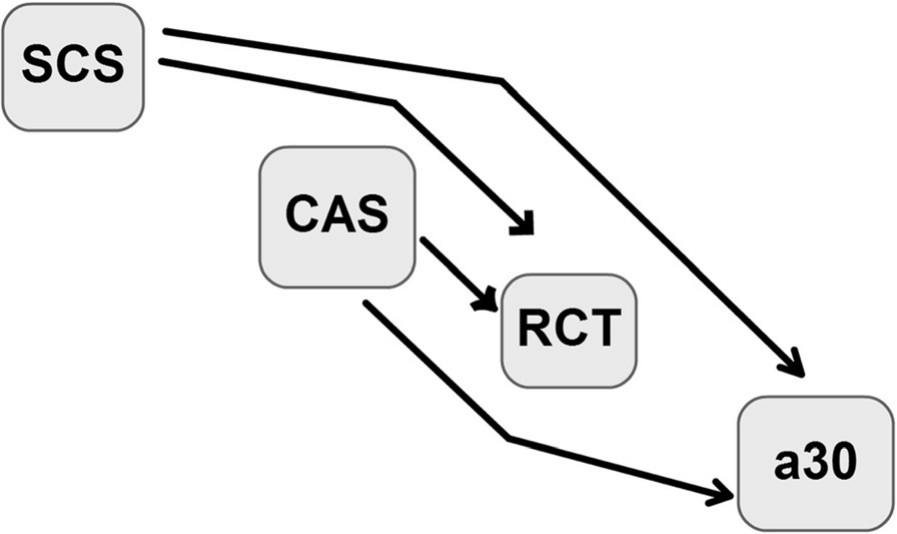
Directed acyclic graph representing the causal structure among phenotypes assigned to model M1. Nodes represent somatic cell score (SCS), casein percentage (CAS), rennet coagulation time after rennet addition (RCT), curd firmness at 30 min after rennet addition (a_30_). The arrows indicate direct causal effects.

Different causal structures with varying complexity in terms of number of causal connections were assigned to each model. Causal connections are represented in the same manner as in Wu et al. [26]. For instance, **λ**_**yx**_ indicates a causal effect of x on y, traits are coded as follows: 1 for SCS, 2 for CAS, 3 for RCT and 4 for a_30_. Model 1 (M1, Figure 1) takes the effect of SCS on RCT and a30 (**λ**_**31**_ and **λ**_**41**_, respectively) and the effect of CAS on RCT and a_30_ (**λ**_**32**_ and **λ**_**42**_, respectively) into account. This model is represented as follows:

$$ \mathbf{M}1\ \left\{\begin{array}{c}\hfill {\mathbf{y}}_1=\mathbf{X}{\mathbf{b}}_1+{\mathbf{Z}}_{\mathbf{h}}{\mathbf{h}}_1+{\mathbf{Z}}_{\mathbf{p}}{\mathbf{p}}_1+{\mathbf{Z}}_{\mathbf{s}}{\mathbf{s}}_1+{\mathbf{e}}_1\hfill \\ {}\hfill {\mathbf{y}}_2=\mathbf{X}{\mathbf{b}}_2+{\mathbf{Z}}_{\mathbf{h}}{\mathbf{h}}_2+{\mathbf{Z}}_{\mathbf{p}}{\mathbf{p}}_2+{\mathbf{Z}}_{\mathbf{s}}{\mathbf{s}}_2+{\mathbf{e}}_2\hfill \\ {}\hfill {\mathbf{y}}_3={\boldsymbol{\uplambda}}_{31}{\mathbf{y}}_1+{\boldsymbol{\uplambda}}_{32}{\mathbf{y}}_2+\mathbf{X}{\mathbf{b}}_3+{\mathbf{Z}}_{\mathbf{h}}{\mathbf{h}}_3+{\mathbf{Z}}_{\mathbf{p}}{\mathbf{p}}_3+{\mathbf{Z}}_{\mathbf{s}}{\mathbf{s}}_3+{\mathbf{e}}_3\hfill \\ {}\hfill {\mathbf{y}}_4={\boldsymbol{\uplambda}}_{41}{\mathbf{y}}_1+{\boldsymbol{\uplambda}}_{42}{\mathbf{y}}_2+\mathbf{X}{\mathbf{b}}_4+{\mathbf{Z}}_{\mathbf{h}}{\mathbf{h}}_4+{\mathbf{Z}}_{\mathbf{p}}{\mathbf{p}}_4+{\mathbf{Z}}_{\mathbf{s}}{\mathbf{s}}_4+{\mathbf{e}}_4\hfill \end{array}\right.. $$

**Figure 2:**
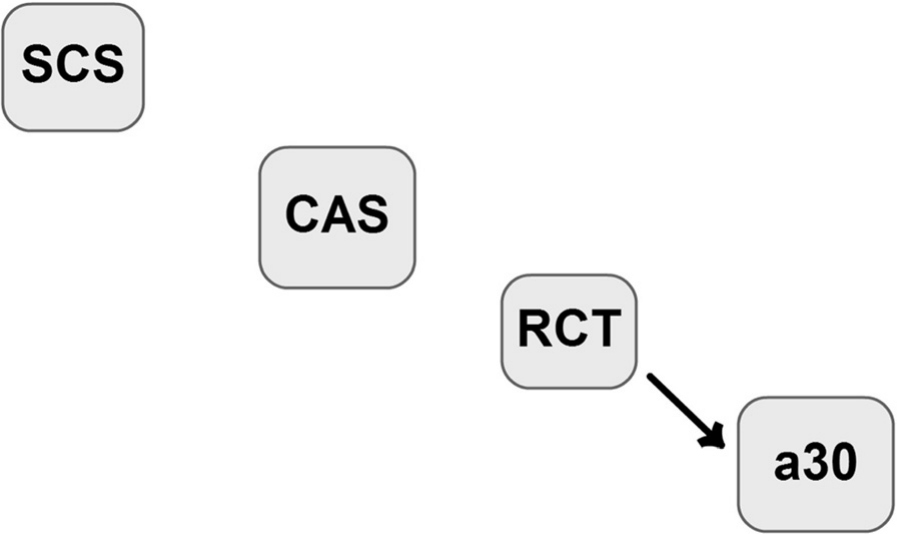
Directed acyclic graph representing the causal structure among phenotypes assigned to model M2. Nodes represent somatic cell score (SCS), casein percentage (CAS), rennet coagulation time after rennet addition (RCT), curd firmness at 30 min after rennet addition (a_30_). The arrows indicate direct causal effects.

In model 2 (M2, Figure 2), only the effect of RCT on a_30_ (**λ**_**43**_) was considered,

$$ \mathbf{M}2\ \left\{\begin{array}{c}\hfill {\mathbf{y}}_1=\mathbf{X}{\mathbf{b}}_1+{\mathbf{Z}}_{\mathbf{h}}{\mathbf{h}}_1+{\mathbf{Z}}_{\mathbf{p}}{\mathbf{p}}_1+{\mathbf{Z}}_{\mathbf{s}}{\mathbf{s}}_1+{\mathbf{e}}_1\hfill \\ {}\hfill {\mathbf{y}}_2=\mathbf{X}{\mathbf{b}}_2+{\mathbf{Z}}_{\mathbf{h}}{\mathbf{h}}_2+{\mathbf{Z}}_{\mathbf{p}}{\mathbf{p}}_2+{\mathbf{Z}}_{\mathbf{s}}{\mathbf{s}}_2+{\mathbf{e}}_2\hfill \\ {}\hfill {\mathbf{y}}_3=\mathbf{X}{\mathbf{b}}_3+{\mathbf{Z}}_{\mathbf{h}}{\mathbf{h}}_3+{\mathbf{Z}}_{\mathbf{p}}{\mathbf{p}}_3+{\mathbf{Z}}_{\mathbf{s}}{\mathbf{s}}_3+{\mathbf{e}}_3\hfill \\ {}\hfill {\mathbf{y}}_4={\boldsymbol{\uplambda}}_{43}{\mathbf{y}}_3+\mathbf{X}{\mathbf{b}}_4+{\mathbf{Z}}_{\mathbf{h}}{\mathbf{h}}_4+{\mathbf{Z}}_{\mathbf{p}}{\mathbf{p}}_4+{\mathbf{Z}}_{\mathbf{s}}{\mathbf{s}}_4+{\mathbf{e}}_4\hfill \end{array}\right.. $$

**Figure 3:**
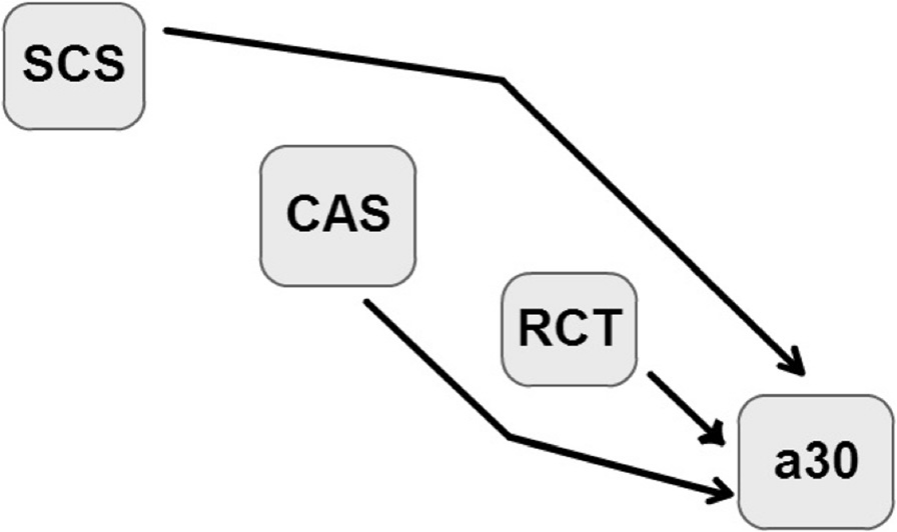
Directed acyclic graph representing the causal structure among phenotypes assigned to model M3. Nodes represent somatic cell score (SCS), casein percentage (CAS), rennet coagulation time after rennet addition (RCT), curd firmness at 30 min after rennet addition (a_30_). The arrows indicate direct causal effects.

In model 3 (M3, Figure 3), effects of SCS, CAS and RCT on a_30_ were considered (**λ**_41,_**λ**_**42**_ and **λ**_**43**_, respectively).

$$ \mathbf{M}3\ \left\{\begin{array}{c}\hfill {\mathbf{y}}_1=\mathbf{X}{\mathbf{b}}_1+{\mathbf{Z}}_{\mathbf{h}}{\mathbf{h}}_1+{\mathbf{Z}}_{\mathbf{p}}{\mathbf{p}}_1+{\mathbf{Z}}_{\mathbf{s}}{\mathbf{s}}_1+{\mathbf{e}}_1\hfill \\ {}\hfill {\mathbf{y}}_2=\mathbf{X}{\mathbf{b}}_2+{\mathbf{Z}}_{\mathbf{h}}{\mathbf{h}}_2+{\mathbf{Z}}_{\mathbf{p}}{\mathbf{p}}_2+{\mathbf{Z}}_{\mathbf{s}}{\mathbf{s}}_2+{\mathbf{e}}_2\hfill \\ {}\hfill {\mathbf{y}}_3=\mathbf{X}{\mathbf{b}}_3+{\mathbf{Z}}_{\mathbf{h}}{\mathbf{h}}_3+{\mathbf{Z}}_{\mathbf{p}}{\mathbf{p}}_3+{\mathbf{Z}}_{\mathbf{s}}{\mathbf{s}}_3+{\mathbf{e}}_3\hfill \\ {}\hfill {\mathbf{y}}_4={\boldsymbol{\uplambda}}_{41}{\mathbf{y}}_1+{\boldsymbol{\uplambda}}_{42}{\mathbf{y}}_2+{\boldsymbol{\uplambda}}_{43}{\mathbf{y}}_3+\mathbf{X}{\mathbf{b}}_4+{\mathbf{Z}}_{\mathbf{h}}{\mathbf{h}}_4+{\mathbf{Z}}_{\mathbf{p}}{\mathbf{p}}_4+{\mathbf{Z}}_{\mathbf{s}}{\mathbf{s}}_4+{\mathbf{e}}_4\hfill \end{array}\right., $$

where **y**_**1**_**, y**_**2**_**, y**_**3**_**, y**_**4**_**, X, b**_**i**_, **Z**_**h**_, **h**_**i**_, **Z**_**p**_, **p**_**i**_, **Z**_**s**_, **s**_**i**_ and **e**_**i**_ are defined as for the MTM. Furthermore, two additional models derived from model M1 were used to avoid possible confounding between the effects of SCS and CAS on RCT and a_30_: one took only the effect of SCS on RCT and a_30_ into account, while the other took only the effect of CAS on RCT and a_30_ into account. However, since the estimated values of the causal effects were similar with model M1 and the two derived models, results from these models are not presented.

Analyses were implemented in a Bayesian framework using the software SIRBAYES [24,26]. For structural coefficients, a multivariate normal prior distribution was assumed as N(**1**λ_0_, **I**τ^2^), where hyperparameters were λ_0_ = 0 and τ^2^ = 10 000. For fixed effects, the prior distribution was normal, with mean 0 and variance 10 000. Prior distributions for sire, cow permanent environmental and herd effects were multivariate normal with the following covariance structures: for sire effect **s ~ N(0, G ⊗ A)** where **A** was the numerator relationship matrix and **G** is the sire effect covariance matrix, and for cow and herd effects and residuals, it was assumed that **p ~ N(0, P ⊗ I)**, **h ~ N(0, H ⊗ I)**, **e ~ N(0, R ⊗ I)** where **I** is an identity matrix and **H**, **P** and **R** are the respective covariance matrices. Prior distributions for covariance matrices **G**, **H**, and **P** were independent inverse-Wishart ***invWish*(ν, S)**, where **ν** are the number of degrees of freedom and **S** is the scale. Prior distribution for **R** was an independent inverse-Wishart ***invWish*(ν, S)** only for the MTM, while an inverted chi-square ***invChisq*(ν, S)** was used for the SEM since **R** was forced to be diagonal. For all priors, the number of degrees of freedom (**ν**) was set to 6. In this study, **R** was assumed as diagonal for SEM, i.e., all residual covariances were constrained to 0. This assumption is required to identify structural coefficients. The meaning of this commonly adopted parametric constraint conflicts with the quantitative genetics that underlie sire MTM, i.e. alleles that are inherited from the dam may have associated effects in more than one trait, and they are expected to be absorbed by the residual covariance. In fact, this theoretical contradiction in SEM that are based on sire models was largely neglected in previous studies [19-31]. However, the possible confounding is not expected to be important here because of the structure of the data and the model: a model that takes into consideration dam effect would not take this effect into account well since most cows do not share the same dam in the dataset.

Structural coefficients were sampled using the Metropolis-Hastings algorithm, and the remaining parameters were sampled using Gibbs sampling [28]. For each model, 120 000 iterations were run, discarding the first 20 000 as burn-in and retaining one every 10 samples for inferences. Posterior means and 95% highest probability density intervals were calculated on the remaining 10 000 samples. Convergence was assessed by visual inspection of the trace and running mean plots and estimates of autocorrelation and effective samples size were obtained using the ‘coda’ package [37] in R (http://cran.r-project.org).

Since the interpretation of the parameters that are estimated with the SEM (contained in **G**, **P**, **H**, and **R**) differs from that of the analogous parameters with a MTM [16], further transformation is required to be able to compare (co)dispersion of overall random effects between the four models fitted. For each model, transformation for the estimated covariance matrices to the MTM scale was performed as:

G* = (**I**-**Λ**)^-1^ **G** (**I**-**Λ**)’^-1^

P* = (**I**-**Λ**)^-1^**P** (**I**-**Λ**)’^-1^

H* = (**I**-**Λ**)^-1^ **H** (**I**-**Λ**)’^-1^

R* = (**I**-**Λ**)^-1^**R** (**I**-**Λ**)’^-1^,

where **G**, **P**, **H**, **R** and **Λ** are defined as above. Genetic and phenotypic correlations were calculated in the usual way from the (co)variance components in G*, P*, H* and R*. Heritability (h^2^) was computed as:

$$ {\mathrm{h}}^2=\frac{4{\upsigma}_{\mathrm{s}}^2}{\upsigma_{\mathrm{s}}^2+{\upsigma}_{\mathrm{p}}^2+{\upsigma}_{\mathrm{h}}^2+{\upsigma}_{\mathrm{e}}^2}, $$

where σ^2^_s_ is the sire additive genetic variance, σ^2^_p_ is the cow permanent environmental variance, σ^2^_h_ is the herd environmental variance and σ^2^_e_ is residual variance. These variance components are obtained from G*, P*, H* and R*. For easier interpretation, posterior means of causal effects were transformed to standard deviation units by applying the formula ${\uplambda}_{yx}^{\prime }={\uplambda}_{yx}\frac{sd(x)}{sd(y)}$, where ${\uplambda}_{yx}^{\prime }$ is the transformed value, λ_*yx*_ is the posterior mean of the causal effect of *x* on *y*, *sd* (*x*) is the standard deviation of the independent variable and *sd* (*y*) is the standard deviation of the dependent variable.

For a_30_, we computed the difference in sire additive genetic and phenotypic variance (sum of sire additive genetic, cow permanent environmental, herd and residual variances) between model M0 and each of the considered models under the causal effect (RCT under model M1 and a_30_ from all models), expressed as the relative difference with model M0 (difference between the variance components divided by the respective variance component of model M0 and scaled to 100). In addition, we computed the heritability from the SEM variance components (i.e., from **G**, **P**, **H** and **R)** according to the formula above.

## Results and discussion

### Descriptive statistics and observed correlations

Descriptive statistics and observed correlations are in Table 1. Means (SD) for SCS, CAS, RCT and a_30_ were equal to 2.35 (1.66), 2.46 (0.23), 18.9 (3.80) and 23.0 (8.53), respectively. The data originated from the same dataset as in Tiezzi et al. [36] but observations were restricted to early-lactation first-parity cows. Descriptive statistics were in partial agreement with the previous study. Summer et al. [38] also found a significant increase in casein percentage from early to late lactation in Italian Friesian cows. Correlations of RCT with SCS and CAS were equal to 0.087 and -0.021, respectively, while correlations of a_30_ with the other traits were -0.849 for RCT, -0.107 for SCS and 0.346 for CAS.

**Table 1:** **Descriptive statistics and observed correlation coefficients for the analyzed traits**

	**Descriptive statistics**				**Correlations**		
**Traits** ^**1**^	**Mean**	**SD**	**Min**	**Max**	**SCS**	**CAS**	**RCT**
SCS	2.35	1.66	-1.32	9.64			
CAS	2.46	0.23	1.68	3.53	0.073		
RCT, min	18.9	3.80	5.52	29.9	0.087	-0.021	
a_30_, mm	23.0	8.53	0.19	54.7	-0.107	0.346	-0.849

^1^Traits are somatic cell score (SCS), casein percentage (CAS), rennet coagulation time (RCT) and curd firmness (a_30_).

### Heritabilities, genetic and phenotypic correlations

Heritabilities (on the diagonal) and genetic and phenotypic correlations (above and below the diagonal, respectively) estimated with each model are in Table 2. These parameter estimators should be interpreted as the standard parameters obtained with a MTM.

**Table 2:** **Estimates** ^1^ **of heritabilities (on the diagonal) genetic (above diagonal) and phenotypic correlations (below diagonal)**

**M0**		**SCS**	**CAS**	**RCT**	**a**_30_
	SCS	**0.030** ^(0.007; 0.059)^	-0.096 ^(-0.515; 0.338)^	-0.081 ^(-0.581; 0.464)^	-0.072 ^(-0.589; 0.476)^
	CAS	0.042 ^(-0.030; 0.117)^	**0.157** ^(0.092; 0.196)^	-0.157 ^(-0.476; -0.182)^	0.374 ^(0.076; 0.654)^
	RCT	0.182 ^(0.108; 0.258)^	-0.046 ^(-0.113; 0.017)^	**0.167** ^(0.083; 0.253)^	-0.918 ^(-0.978; -0.846)^
	a_30_	-0.192 ^(-0.264; -0.117)^	0.291 ^(0.230; 0.348)^	-0.851 ^(-0.872; -0.830)^	**0.187** ^(0.094; 0.287)^
**M1**		SCS	CAS	RCT	a_30_
	SCS	**0.021** ^(0.005; 0.042)^	0.059 ^(-0.342; 0.438)^	0.081 ^(-0.457; 0.599)^	-0.090 ^(-0.570; 0.410)^
	CAS	0.040 ^(-0.019; 0.100)^	**0.141** ^(0.083; 0.205)^	-0.155 ^(-0.466; 0.162)^	0.474 ^(0.226; 0.716)^
	RCT	0.152 ^(0.095; 0.208)^	-0.125 ^(-0.181; -0.069)^	**0.163** ^(0.095; 0.236)^	-0.933 ^(-0.974; -0.885)^
	a_30_	-0.158 ^(-0.216; -0.106)^	0.494 ^(0.451; 0.537)^	-0.580 ^(-0.610; -0.548)^	**0.171** ^(0.102; 0.250)^
**M2**		SCS	CAS	RCT	a_30_
	SCS	**0.025** ^(0.007; 0.049)^	-0.180 ^(-0.599; 0.277)^	-0.031 ^(-0.532; 0.497)^	-0.149 ^(-0.669; 0.348)^
	CAS	0.021 ^(-0.039; 0.081)^	**0.144** ^(0.082; 0.217)^	-0.241 ^(-0.568; 0.096)^	0.481 ^(0.205; 0.726)^
	RCT	0.152 ^(0.097; 0.207)^	-0.048 ^(-0.100; 0.002)^	**0.116** ^(0.058; 0.179)^	-0.911 ^(-0.978; -0.830)^
	a_30_	-0.151 ^(-0.208; -0.096)^	0.211 ^(0.161; 0.259)^	-0.841 ^(-0.856; -0.826)^	**0.139** ^(0.072; 0.219)^
**M3**		SCS	CAS	RCT	a_30_
	SCS	**0.029** ^(0.007; 0.056)^	0.050 ^(-0.330; 0.437)^	-0.001 ^(-0.505; 0.495)^	-0.080 ^(-0.556; 0.382)^
	CAS	0.035 ^(-0.021; 0.095)^	**0.143** ^(0.083; 0.208)^	-0.162 ^(-0.487; 0.150)^	0.572 ^(0.345; 0.784)^
	RCT	0.147 ^(0.094; 0.204)^	-0.021 ^(-0.070; -0.027)^	**0.112** ^(0.055; 0.172)^	-0.883 ^(-0.953; -0.804)^
	a_30_	-0.163 ^(-0.219; -0.109)^	0.463 ^(0.421; 0.502)^	-0.804 ^(-0.821; -0.787)^	**0.142** ^(0.077; 0.214)^

^1^Estimates are the means (lower and upper bound of the 95% HPD interval) of the marginal posterior distributions^.^. Models differ in the structural coefficients considered: M0 is the standard multiple trait model; in M1 are considered the causal effects of both SCS and CAS on RCT and a_30_; in M2 is considered the causal effects of RCT on a_30_; in M3 the causal effects of SCS, CAS and RCT on a_30_ are considered.

Heritabilities for all traits considered were consistent across models: SCS ranged from 0.021 in model M1 to 0.030 in model M0, CAS ranged from 0.141 in M1 to 0.157 in M2, RCT from 0.112 in M3 to 0.167 in M0 and a_30_ ranged from 0.139 in M2 to 0.187 in M0. Also, genetic and phenotypic correlations did not vary significantly across models, since in most cases, the posterior mean of one model fell within the 95% HPD (highest posterior density interval) intervals of the other models. Genetic correlations that involved RCT were almost null with SCS (posterior means ranging from -0.081 to 0.081), very moderate and negative with CAS (-0.241 to -0.155), and strong and negative with a_30_ (-0.933 to -0.883). The trait a_30_ presented weak negative correlations with SCS (-0.149 to -0.072) and moderate positive correlations with CAS (0.374 to 0.572). Genetic correlation between SCS and CAS was null, ranging from -0.180 to 0.059 across models. Phenotypic and genetic correlations were similar in direction and magnitude: RCT was moderately and positively correlated with SCS (0.147 to 0.182) and strongly and negatively correlated with a_30_ (-0.851 to -0.58), while correlation with CAS was null (-0.125 to -0.021). a_30_ was weakly and negatively correlated with SCS (-0.192 to -0.151) and moderately and positively correlated with CAS (0.211 to 0.494), while correlation between SCS and CAS was null (0.021 to 0.042).

Estimates of heritabilities obtained with the MTM were lower than those reported by Tiezzi et al. [36], who considered whole lactations up to the ninth parity. In our study, restricting the dataset to early-lactation first-parity cows led to decreased heritabilities for all traits: from 0.093 to 0.030 for SCS, from 0.283 to 0.157 for CAS, from 0.210 to 0.167 for RCT and from 0.238 to 0.187 for a_30_. To the best of our knowledge, there are no studies on the heterogeneity of variance components across lactation and parities for milk coagulation properties; however, Muir et al. [39] found a lower heritability for SCS in the first lactation than in later lactations for Italian Holsteins (0.165, 0.211 and 0.252 for first, second and third lactations, respectively), while Odegard et al. [40] reported a heritability for SCS less than 0.08 at the beginning of lactation and a value of 0.10 in late lactation, although they used Norwegian Red cattle data. Estimates of genetic and phenotypic correlations are in agreement with Tiezzi et al. [36], therefore stage and number of lactation do not appear to affect correlations.

As mentioned above, we found no significant differences in estimates of genetic and phenotypic correlations between MTM and SEM. However, exceptions were observed between CAS and a_30_, i.e., models M0 and M2 led to lower values (0.291 and 0.211, respectively) while models M1 and M3 led to the highest values (0.494 and 0.463, respectively). Including causal effects between CAS and a_30_ increased the correlation estimates. This was observed in several other studies: (1) Konig et al. [29] who analyzed correlations between milk yield and claw disorders in German Holstein, found that SEM resulted in lower genetic correlations compared to MTM; (2) de los Campos et al. [21] reported that, in dairy goats, genetic correlations between milk yield and SCS differed between MTM and SEM; and (3) Wu et al. [26] showed that including causal effects modified the genetic correlations between milk yield and SCS, while heritabilities varied little across models.

### Causal effects on RCT and a_30_

The causal effects estimated with the three SEM and their transformations to the scale of standard deviation units for both traits are in Table 3. Model M1 took the effects of traditional milk quality parameters (SCS and CAS) on milk coagulation measures (RCT and a_30_) into account. According to the posterior mean of this parameter, an increase of 1 unit in SCS (e.g. from 2.00 to 3.00) causally increased RCT by 0.242 min (0.1057 SD units increase in RCT per 1 SD unit increase in SCS). The value ‘0’ was not included in the 95% HPD intervals (0.196 to 0.288). However, an increase of 1 unit in CAS (e.g. from 2% to 3%) decreased RCT by 3.043 min (95% HPD intervals: -3.372 to -2.705, -0.1842 SD units). Similarly, the impact of the same variables on a_30_ was as follows: an increase of 1 unit in SCS led to a 0.703 mm reduction in a_30_ (95% HPD intervals: -0.824 to -0.625. -0.1421 SD units) and an increase of 1 unit in CAS led to a 18.823 mm increase in a_30_ (95% HPD intervals: 18.128 to 19.595, 0.5075 SD units).

**Table 3:** **Estimates** ^**1**^ **of causal effects with different models** ^**2**^ **and transformation to standard deviation units** ^**3**^

**Causal effect**	**M1**		**M2**		**M3**	
	**Estimate**	**SD Units**	**Estimate**	**SD Units**	**Estimate**	**SD Units**
SCS - > RCT	0.242 ^(0.196; 0.288)^	0.1057	.	.	.	.
CAS - > RCT	-3.043 ^(-3.372; -2.705)^	-0.1842	.	.	.	.
SCS - > a_30_	-0.730 ^(-0.824; -0.625)^	-0.1421	.	.	-0.267 ^(-0.327; 0.207)^	-0.0520
CAS - > a_30_	18.823 ^(18.128; 19.595)^	0.5075	.	.	12.845 ^(12.443; 13.232)^	0.3465
RCT - > a_30_	.	.	-1.901 ^(-1.931; -1.869)^	-0.8469	-1.792 ^(-1.819; -1.764)^	-0.7983

^1^Estimates are the means (lower and upper bound of the 95% HPD interval) of the marginal posterior distributions; ^2^the models differ in the causal effects considered: M0 is the standard multiple trait model; in M1 are considered the causal effects of both SCS and CAS on RCT and a_30_; in M2 is considered the causal effects of RCT on a_30_; in M3 the causal effects of SCS, CAS and RCT on a_30_ are considered; ^3^causal effects were transformed to standard deviation units by applying the formula ${\uplambda}_{yx}^{\prime }={\uplambda}_{yx}\frac{sd(x)}{sd(y)}$, where ${\uplambda}_{yx}^{\prime }$ is the transformed value, λ_*yx*_ is the posterior mean of the causal effect of *x* on *y*, *sd* (*x*) is the standard deviation of the independent variable and *sd* (*y*) is the standard deviation of the dependent variable.

Fitting model M2 resulted in a decrease of the posterior mean of 1.901 mm in a_30_ per min increase in RCT (95% HPD intervals: -1.931 to -1.869, -0.8469 SD units).

The effects inferred from model M3 were weaker than those from other models, although they agreed in sign. The posterior means of the effect of SCS, CAS and RCT on a_30_ were equal to -0.267 (95% HPD intervals: -0.327 to 0.207, -0.0520 SD units), 12.845 (95% HPD intervals: 12.443 to 13.232, 0.3465 SD units) and -1.792 (95% HPD intervals: -1.819 to -1.764, -0.7983 SD units), respectively.

Overall, increasing milk quality (i.e. lower SCS and higher CAS) led to better milk coagulation ability (i.e. lower RCT and greater a_30_), although the traits used and the strength of the relationship varied across studies. Reviewing the effects of SCS on cheese process and quality, Le Marechal et al. [14] found that, in most of the studies, a high SCS was associated with extended rennet clotting time and lower curd firmness, and Mazal et al. [41] showed that curd firmness of milk decreased as SCS decreased from ~ 800 000 to 170 000 cells per mL). Politis and Ng-Kwai-Hang [13] reported a regression coefficient of a_30_ on CAS of 12.92, which is very close to the value found here (12.845) although the methodologies used differ and the required parametric interpretation does not allow straightforward comparisons. However, in a study conducted by Grandison and Ford [12], a correlation of 0.807 was estimated between SCC and coagulum strength.

### Impact of causal effects on curd firmness

Table 4 shows the genetic and phenotypic variances and the estimated heritabilities, for a_30_ when causal effect(s) between phenotypes are not accounted for (i.e., excluding random effects mediated by other traits). These parameters express the dispersion of random effects that affect a_30_ directly, i.e., not mediated by other phenotypic traits according to each SEM fitted. Here, the genetic variance and heritability that only account for direct effects (i.e., under a scenario in which the other traits were physically maintained at a constant value) can be compared with the standard, overall genetic variance and heritability estimated from a classic MTM. Sire additive genetic variance decreased as the number of traits considered to have a causal effect on a_30_ increased i.e. to 3.829 with the baseline MTM M0 model and to 3.135 (instead of the initial 18,1% variance) with model M1 in which both causal effects of SCS and CAS were taken into account, strongly decreased to 0.536 with model M2 (-86.0% of the variance with M0) due to the sole effect of RCT, and finally decreased to 0.128 with model M3, for which 96.7% of the overall sire additive genetic variance inferred from model M0 was absorbed by the causal effects of SCS, CAS or RCT. A similar trend was observed for the phenotypic variance, which decreased from 81.998 with M0 to 70.192 with M1 (-14.4%), 22.108 with M2 (-73.1%) and to 11.902 with M3 (-85.5%). It seems that most of the variance of a_30_ was assigned to a path mediated by RCT, which reflects that curd firmness depends on when coagulation starts during the test. If the starting time of the coagulation was hypothetically set at the same value for all milk samples through external interventions (scenario under model M2), only 26.9% of the total observed variability remains for a_30_. The decrease in sire additive genetic variance was larger than phenotypic variance, which resulted in a decreased heritability for a_30_ from 0.187 with model M0, to 0.179 with M1, 0.097 with M2 and 0.043 with M3.

**Table 4:** **Estimates** ^**1**^ **of variance components for a**_**30**_ **when influence of causal effects is removed**

	**σ** ^2^_s_	**Δσ** ^2^_s_ **(%)**	**σ** ^2^_y_	**Δσ** ^2^_y_ **(%)**	**h** ^2^
M0	3.829 ^(1.964; 5.999)^	-	81.998 ^(73.390; 91.287)^	-	0.187 ^(0.094; 0.287)^
M1	3.135 ^(1.774; 4.528)^	-18.1	70.192 ^(65.051; 75.771)^	-14.4	0.179 ^(0.107; 0.259)^
M2	0.536 ^(0.219; 0.895)^	-86.0	22.108 ^(20.453; 23.780)^	-73.1	0.097 ^(0.040; 0.160)^
M3	0.128 ^(0.044; 0.212)^	-96.7	11.902 ^(11.324; 12.488)^	-85.5	0.043 ^(0.016; 0.073)^

Parameters reported are sire additive genetic variance (σ2s) and phenotypic variance2 (σ2y), and relative losses (Δσ2s and Δσ2y, respectively) from the baseline multiple trait model (M0) for the models3 considered.

^1^Estimates are the means (lower and upper bound of the 95% HPD interval) of the marginal posterior distributions; ^2^the phenotypic variance is considered as sum of the sire additive genetic, cow permanent environmental, herd and residual components; ^3^the models differ in the causal effects considered: M0 is the standard multiple trait model; in M1 are considered the causal effects of both SCS and CAS on RCT and a_30_; in M2 is considered the causal effects of RCT on a_30_; in M3 the causal effects of SCS, CAS and RCT on a_30_ are considered.

### Causal relationships between somatic cell score, casein percentage and milk coagulation properties

Our results suggest that even under a hypothetical scenario in which SCS and CAS are maintained at constant values by external intervention and their influence on variability is nullified, the firming process is expected to vary, with part of the variability being attributable to the additive genetic component. In an experimental scenario in which CAS and SCS are standardized across samples, we would still find some additive genetic variation in RCT and a_30_. However, the causal pattern appears to differ between RCT and a_30_.

For RCT, both direct sire additive genetic and phenotypic variances obtained with model M1 were essentially equal to those obtained with the MTM scenario (results reported in Table S2 [See Additional file 1: Table S2]), which is what would be expected if variation in RCT was weakly mediated by traditional milk quality parameters. In fact, RCT showed low phenotypic correlations with SCS and CAS, which was translated as weak inferred causal effects, especially from CAS. Weak statistical dependences generally suggest the absence of strong causal effects.

Curd firmness can be considered as the most pertinent coagulation measure that can account for cheese yield under certain processing conditions [7]. Assuming model M1, 18.1% of the additive genetic variance and 14.4% of the phenotypic variance can be explained by causal effects of SCS and CAS, which leaves a large proportion of the variation explained by other sources. The moderate correlations between a_30_ and CAS inferred with MTM (an observed phenotypic correlation of 0.346 (Table 1) and a genetic correlation of 0.374 (Table 2)) can be considered to result from a causal effect rather than from a common source of variation i.e., the difference in variability for a_30_ between real and causality-free scenarios was noticeable for all variance components but relatively larger for the additive genetic variance. More evidence is provided by the fact that the square of the phenotypic and genetic correlations between a_30_ and CAS is close to the drop in variance for a_30_ under model M2 (Table 4). This scenario suggests that the overall genetic association, which is observed between CAS and a_30_ with MTM, is mostly due to a phenotypic causal effect of the former on the latter, rather than a pleiotropic effect on both traits directly. This hypothesis is supported by the null values estimated for genetic covariances under model M1 [See Additional file 1: Table S1].

Here, we found causal dependencies between RCT and a_30_. Our results confirm that curd firmness is intrinsically connected with RTC and depends causally on it [10], which suggests that it virtually cannot vary when the latter is held constant. In this study, model M2 accounted for this dependency as an effect of RCT on a_30_. This type of model makes it possible to investigate how the system would react if the samples were physically set to have the same coagulation time, i.e. removing variability due to the effect of starting coagulation time. Variance components and heritabilities for scenarios that involve such interventions can be inferred from information provided by **Λ, G**, **P**, **H** and **R** pertaining to model M2 before transformation to the standard MTM, and are in Table 4. It should be noted that the heritability of a_30_ (0.097) is low, but non-null, and the posterior mean (95% HDP intervals) of its genetic covariance with CAS is 0.030 (0.011; 0.052), similar to the corresponding genetic covariance of 0.052 (0.001; 0.105) obtained with model M0 [See Additional file 1: Table S1]. These results indicate that the association between the genetic effect of CAS and the genetic effect of a_30_ that is not mediated by RCT is still present. Thus, under model M2, curd firmness can be considered as a trait by itself and should not be ignored in selection indices, despite its low heritability (0.097), which will constrain genetic progress.

The almost complete loss of variance for a_30_ with model M3 suggests that the causal effects of SCS, CAS and RCT absorb a large part of the phenotypic variation of a_30_. Subsequently, independent sources of environmental variation are scarce and direct additive genetic variance is negligible. Under the causal assumptions applied here, we show that the observed genetic associations between a_30_ and the other traits are due to phenotypic causal effects rather than to common sources of genetic variation, i.e. genes with pleiotropic effects. Under these circumstances, the importance of a_30_ in selection indices should be downplayed if SCS, CAS and RCT are already taken into account.

## Conclusions

This study inferred causal relationships between two traditional milk quality measures (somatic cell score and casein percentage) and milk coagulation properties (rennet coagulation time and curd firmness). Results from this study suggest that the additive genetic variance of milk coagulation properties does not depend on traits such as somatic cell score or casein percentage. If selection is performed on these traits, coagulation properties will be improved only indirectly and to a small extent. This means that selection for milk coagulation properties should be performed on their direct measures, and cannot rely entirely on correlated traits since external interventions on the correlated traits may break down the causal path. In addition, including both rennet coagulation time and curd firmness in genetic evaluations appears redundant, considering that the latter depends largely on the former. If rennet coagulation time is the only selection objective, specific models that include right-censoring would probably better suit this purpose, because 5 to 10% samples of milk do not start coagulation within the first 30 min after rennet addition. Otherwise, it is necessary to demonstrate that curd firmness can have an impact on cheese yield and quality of products even if coagulation time is physically set to a constant value. This trait could represent an additional selection objective, but further research is needed.

## Additional file

Additional file 1: Table S1. Estimates^1^ of co-variance components for sire additive genetic effect, cow permanent environmental effect, herd effect and residual as estimated with different models^2^. ^1^Estimates are the means (lower and upper bound of the 95% HPD interval) of the marginal posterior distributions.^2^ The models differ in the structural coefficients considered: M0 is the standard multiple trait model; in M1 are considered the causal effects of both SCS and CAS on RCT and a_30_; in M2 is considered the causal effects of RCT on a_30_; in M3 the causal effects of SCS, CAS and RCT on a_30_ are considered.
