# Supplementary material for: Causal relationships between milk quality and coagulation properties in Italian Holstein-Friesian dairy cattle
Source: Genet Sel Evol. 2015 May 13;47(1):45. doi: 10.1186/s12711-015-0123-7 (PMC4429925; doi:10.1186/s12711-015-0123-7)
Supplement: Additional file 1: Table S1. — Estimates1 of co-variance components for sire additive genetic effect, cow permanent environmental effect, herd effect and residual as estimated with different models2. 1Estimates are the means (lower and upper bound of the 95% HPD interval) of the marginal posterior distributions.2 The models differ in the structural coefficients considered: M0 is the standard multiple trait model; in M1 are considered the causal effects of both SCS and CAS on RCT and a30; in M2 is considered the causal effects of RCT on a30; in M3 the causal effects of SCS, CAS and RCT on a30 are considered. [file 12711_2015_123_MOESM1_ESM.pdf]

Table S1: Estimates of co-variance components for sire additive genetic effect, cow permanenet environmental effect, herd effect and residual as estimated with model 0 (M0).

Sire Additive Genetic Effect

	SCS	CAS	RCT	a30
SCS	0.037 (0.010; 0.072)	-0.001 (-0.008; 0.005)	-0.012 (-0.096; 0.068)	-0.028 (-0.234; 0.192)
CAS	-0.001 (-0.008; 0.005)	0.005 (0.003; 0.007)	-0.009 (-0.029; 0.010)	0.052 (0.001; 0.105)
RCT	-0.012 (-0.096; 0.068)	-0.009 (-0.029; 0.010)	0.602 (0.302; 0.936)	-1.397 (-2.179; -0.678)
a30	-0.028 (-0.234; 0.192)	0.052 (0.001; 0.105)	-1.397 (-2.179; -0.678)	3.829 (1.964; 5.999)

Cow Permanent Environmental Effect

	SCS	CAS	RCT	a30
SCS	2.143 (1.610; 2.709)	0.013 (-0.042; 0.069)	0.918 (0.304; 1.611)	-2.159 (-3.723; -0.665)
CAS	0.013 (-0.042; 0.069)	0.065 (0.053; 0.078)	0.008 (-0.077; 0.090)	0.209 (0.012; 0.418)
RCT	0.918 (0.304; 1.611)	0.008 (-0.077; 0.090)	3.778 (2.309; 5.279)	-7.553 (-10.860; -4.238)
a30	-2.159 (-3.723; -0.665)	0.209 (0.012; 0.418)	-7.553 (-10.860; -4.238)	20.451 (12.077; 29.057)

Herd Effect

	SCS	CAS	RCT	a30
SCS	1.403 (1.256; 1.540)	0.005 (-0.009; 0.017)	0.356 (0.170; 0.549)	-1.004 (-1.454; -0.541)
CAS	0.005 (-0.009; 0.017)	0.033 (0.030; 0.036)	-0.001 (-0.026; 0.023)	0.290 (0.226; 0.352)
RCT	0.356 (0.170; 0.549)	-0.001 (-0.026; 0.023)	3.751 (3.293; 4.258)	-8.170 (-9.347; -7.158)
a30	-1.004 (-1.454; -0.541)	0.290 (0.226; 0.352)	-8.170 (-9.347; -7.158)	22.886 (20.213; 25.893)

Residual

	SCS	CAS	RCT	a30
SCS	1.305 (1.256; 1.354)	0.016 (0.011; 0.020)	0.273 (0.197; 0.352)	-0.648 (-0.830; -0.469)
CAS	0.016 (0.011; 0.020)	0.021 (0.020; 0.021)	-0.059 (-0.069; -0.050)	0.375 (0.351; 0.400)
RCT	0.273 (0.197; 0.352)	-0.059 (-0.069; -0.050)	6.313 (6.086; 6.551)	-12.165 (-12.678; -11.661)
a30	-0.648 (-0.830; -0.469)	0.375 (0.351; 0.400)	-12.165 (-12.678; -11.661)	34.832 (33.518; 36.130)

Table S2: Co-variance components for sire additive genetic effect, cow permanenet environmental effect, herd effect and residual as estimated with model 1 (M1).

Sire Additive Genetic Effect

	SCS	CAS	RCT	a30
SCS	0.020 (0.005; 0.039)	0.001 (-0.003; 0.004)	0.006 (-0.056; 0.071)	-0.021 (-0.162; 0.117)
CAS	0.001 (-0.003; 0.004)	0.003 (0.002; 0.005)	0.003 (-0.013; 0.018)	-0.006 (-0.042; 0.026)
RCT	0.006 (-0.056; 0.071)	0.003 (-0.013; 0.018)	0.631 (0.370; 0.929)	-1.393 (-2.034; -0.846)
a30	-0.021 (-0.162; 0.117)	-0.006 (-0.042; 0.026)	-1.393 (-2.034; -0.846)	3.135 (1.774; 4.528)

Cow Permanent Environmental Effect

	SCS	CAS	RCT	a30
SCS	1.284 (0.973; 1.616)	0.013 (-0.022; 0.048)	0.315 (-0.130; 0.741)	-0.654 (-1.564; 0.265)
CAS	0.013 .018 (-0.022; 0.048)	0.044 (0.036; 0.052)	0.062 (-0.003; 0.126)	-0.123 (-0.261; 0.011)
RCT	0.315 (-0.130; 0.741)	0.062 (-0.003; 0.126)	4.428 (3.328; 5.511)	-8.716 (-10.743; -6.747)
a30	-0.654 (-1.564; 0.265)	-0.123 (-0.261; 0.011)	-8.716 (-10.743; -6.747)	18.793 (14.098; 23.784)

Herd Effect

	SCS	CAS	RCT	a30
SCS	0.841 (0.755; 0.921)	0.011 (0.003; 0.018)	0.021 (-0.114; 0.153)	-0.035 (-0.323; 0.259)
CAS	0.011 (0.003; 0.018)	0.021 (0.019; 0.022)	0.063 (0.045; 0.080)	-0.137 (-0.177; -0.098)
RCT	0.021 (-0.114; 0.153)	0.063 (0.045; 0.080)	4.344 (4.009; 4.693)	-9.431 (-10.120; -8.755)
a30	-0.035 (-0.323; 0.259)	-0.137 (-0.177; -0.098)	-9.431 (-10.120; -8.755)	20.941 (19.270; 22.627)

Residual

	SCS	CAS	RCT	a30
SCS	1.631 (1.565; 1.690)	0	0	0
CAS	0	0.025 (0.024; 0.026)	0	0
RCT	0	0	6.044 (5.854; 6.238)	0
a30	0	0	0	27.323 (26.471; 28.241)

Structural Coefficients

	SCS	CAS	RCT	a30
SCS	-	0	0	0
CAS	0	-	0	0
RCT	0.242 (0.196; 0.288)	-3.043 (-3.372; -2.705)	-	0
a30	-0.730 (-0.824; -0.625)	18.823 (18.128; 19.595)	0	-

Table S3: Co-variance components for sire additive genetic effect, cow permanenet environmental effect, herd effect and residual as estimated with model 2 (M2).

Sire Additive Genetic Effect

	SCS	CAS	RCT	a30
SCS	0.024 (0.006; 0.046)	-0.002 (-0.007; 0.003)	-0.003 (-0.054; 0.049)	-0.044 (-0.117; 0.022)
CAS	-0.002 (-0.007; 0.003)	0.004 (0.002; 0.005)	-0.009 (-0.023; 0.004)	0.030 (0.011; 0.052)
RCT	-0.003 (-0.054; 0.049)	-0.009 (-0.023; 0.004)	0.370 (0.186; 0.580)	-0.195 (-0.398; 0.003)
a30	-0.044 (-0.117; 0.022)	0.030 (0.011; 0.052)	-0.195 (-0.398; 0.003)	0.536 (0.219; 0.895)

Cow Permanent Environmental Effect

	SCS	CAS	RCT	a30
SCS	1.298 (0.960; 1.614)	0.007 (-0.028; 0.044)	0.694 (0.297; 1.082)	-0.295 (-0.815; 0.208)
CAS	0.007 (-0.028; 0.044)	0.046 (0.038; 0.055)	-0.017 (-0.070; 0.037)	0.190 (0.110; 0.269)
RCT	0.694 (0.297; 1.082)	-0.017 (-0.070; 0.037)	2.202 (1.365; 3.110)	-0.704 (-1.565; 0.142)
a30	-0.295 (-0.815; 0.208)	0.190 (0.110; 0.269)	-0.704 (-1.565; 0.142)	4.023 (2.483; 5.589)

Herd Effect

	SCS	CAS	RCT	a30
SCS	0.846 (0.763; 0.931)	0.007 (-0.001; 0.015)	0.368 (0.266; 0.473)	-0.205 (-0.338; -0.073)
CAS	0.007 (-0.001; 0.015)	0.025 (0.023; 0.026)	-0.028 (-0.043; -0.014)	0.251 (0.228; 0.273)
RCT	0.368 (0.266; 0.473)	-0.028 (-0.043; -0.014)	2.149 (1.852; 2.426)	-0.894 (-1.165; -0.629)
a30	-0.205 (-0.338; -0.073)	0.251 (0.228; 0.273)	-0.894 (-1.165; -0.629)	4.442 (3.979; 4.908)

Residual

	SCS	CAS	RCT	a30
SCS	1.628 (1.568; 1.691)	0	0	0
CAS	0	0.024 (0.023; 0.025)	0	0
RCT	0	0	7.995 (7.704; 8.292)	0
a30	0	0	0	13.107 (12.695; 13.550)

Structural Coefficients

	SCS	CAS	RCT	a30
SCS	-	0	0	0
CAS	0	-	0	0
RCT	0	0	-	0
a30	0	0	-1.901 (-1.931; -1.869)	-

Table S4: Co-variance components for sire additive genetic effect, cow permanenet environmental effect, herd effect and residual as estimated with model 3 (M3).

Sire Additive Genetic Effect

	SCS	CAS	RCT	a30
SCS	0.027 (0.007; 0.053)	0.000 (-0.003; 0.004)	-0.000 (-0.053; 0.049)	-0.022 (-0.059; 0.011)
CAS	0.000 (-0.003; 0.004)	0.003 (0.002; 0.005)	-0.006 (-0.018; 0.006)	0.002 (-0.005; 0.010)
RCT	-0.000 (-0.053; 0.049)	-0.006 (-0.018; 0.006)	0.361 (0.187; 0.567)	-0.164 (-0.270; -0.062)
a30	-0.022 (-0.059; 0.011)	0.002 (-0.005; 0.010)	-0.164 (-0.270; -0.062)	0.128 (0.044; 0.212)

Cow Permanent Environmental Effect

	SCS	CAS	RCT	a30
SCS	1.294 (0.966; 1.621)	0.011 (-0.023; 0.045)	0.679 (0.296; 1.075)	-0.122 (-0.473; 0.216)
CAS	0.011 (-0.023; 0.045)	0.044 (0.036; 0.052)	-0.003 (-0.056; 0.046)	0.006 (-0.026; 0.039)
RCT	0.679 (0.296; 1.075)	-0.003 (-0.056; 0.046)	2.364 (1.456; 3.247)	-0.650 (-1.200; -0.108)
a30	-0.122 (-0.473; 0.216)	0.006 (-0.026; 0.039)	-0.650 (-1.200; -0.108)	0.835 (0.393; 1.335)

Herd Effect

	SCS	CAS	RCT	a30
SCS	0.839 (0.752; 0.922)	0.010 (0.002; 0.018)	0.352 (0.249; 0.457)	-0.067 (-0.178; 0.049)
CAS	0.010 (0.002; 0.018)	0.021 (0.019; 0.022)	-0.014 (-0.028; -0.001)	0.001 (-0.011; 0.013)
RCT	0.352 (0.249; 0.457)	-0.014 (-0.028; -0.001)	2.287 (2.015; 2.575)	-0.919 (-1.115; -0.727)
a30	-0.067 (-0.178; 0.049)	0.001 (-0.011; 0.013)	-0.919 (-1.115; -0.727)	0.981 (0.806; 1.160)

Residual

	SCS	CAS	RCT	a30
SCS	1.631 (1.569; 1.693)	0	0	0
CAS	0	0.025 (0.024; 0.026)	0	0
RCT	0	0	7.872 (7.596; 8.166)	0
a30	0	0	0	9.958 (9.635; 10.291)

Structural Coefficients

	SCS	CAS	RCT	a30
SCS	-	0	0	0
CAS	0	-	0	0
RCT	0	0	-	0
a30	-0.267 (-0.327; -0.207)	12.845 (12.443; 13.232)	-1.792 (-1.819; -1.764)	-
